# Investigation of hepatitis E virus seroprevalence and risk factors in hemodialysis patients

**DOI:** 10.3389/fpubh.2025.1574361

**Published:** 2025-05-09

**Authors:** Hakkı Öztürk, Metin Özsoy, Ayşegül Tuna, Artuner Varlıbaş, Salih Cesur, Altan Aksoy, Aydın Çifci, Mehmet Emin Demir

**Affiliations:** ^1^Private Balgat Dialysis Center, Hemodialysis Physician, Specialist in Infectious Disease Epidemiology, Ankara, Türkiye; ^2^Ankara Training and Research Hospital, Department of Infectious Diseases and Clinical Microbiology, Health Sciences University, Ankara, Türkiye; ^3^Faculty of Medicine, Department of Infectious Diseases and Clinical Microbiology, Kırıkkale University, Kırıkkale, Türkiye; ^4^Faculty of Medicine, Department of Internal Medicine, Kırıkkale University, Kırıkkale, Türkiye; ^5^Department of Medical Microbiology, Ankara Bilkent City Hospital, Ankara, Türkiye; ^6^Atılım University, Ankara, Türkiye

**Keywords:** hepatitis E virus, hemodialysis, seroprevalence, risk factors, immunosuppression

## Abstract

**Background:**

Hemodialysis patients are at increased risk for hepatitis E virus (HEV) infection due to their immunocompromised status and frequent exposure to invasive medical procedures. HEV can lead to chronic infections and severe complications, particularly in high-risk populations. This study aimed to determine HEV-IgG seroprevalence among hemodialysis patients in Ankara, Turkey, and evaluate associated risk factors.

**Methods:**

A total of 160 hemodialysis patients from three private dialysis centers in Ankara were included in this prospective, cross-sectional study. Anti-HEV-IgG antibodies were detected using the ELISA method. Demographic characteristics and potential risk factors, including dialysis duration, comorbidities, blood transfusion history, drinking water source, dietary habits, and involvement in animal husbandry, were assessed via structured surveys. Statistical analyses were conducted using SPSS Version 22.0, with Pearson’s chi-square and Fisher’s exact tests applied to categorical variables. Logistic regression analysis was performed to identify independent risk factors for HEV seropositivity.

**Results:**

HEV-IgG seropositivity was detected in 42 patients (26.25%). Seroprevalence increased significantly with age, rising from 6.7% in patients under 55 years to 47.4% in those over 65 years (*p* < 0.001). Extended dialysis duration (>5 years) was also significantly associated with HEV seropositivity (*p* = 0.02). However, no significant associations were found between HEV seropositivity and gender, blood transfusion history, source of drinking water, consumption of raw meat, or involvement in animal husbandry (*p* > 0.05).

**Conclusion:**

The HEV-IgG seroprevalence among hemodialysis patients in Ankara was higher than previously reported rates in Turkey. Age and prolonged dialysis duration emerged as significant risk factors, underscoring the importance of screening and preventive strategies in this vulnerable population. Further multi-regional studies are needed to better understand HEV transmission dynamics and improve management strategies in hemodialysis patients.

## Introduction

Hepatitis E virus (HEV) is a globally prevalent, non-enveloped RNA virus from the Hepeviridae family and one of the five primary causes of viral hepatitis (1). While it is typically transmitted via the fecal-oral route—often through contaminated food or inadequate sanitation—it can also spread through blood transfusion, parenteral exposure, organ transplantation, and vertical transmission (1, 2). Although HEV infections are generally self-limiting in healthy individuals, they can pose serious risks for immunocompromised populations. Among these, hemodialysis patients are particularly vulnerable due to frequent invasive procedures and impaired immune function (3, 4). Despite growing awareness of HEV’s clinical relevance, data on its seroprevalence and associated risk factors in this high-risk group remain limited. A better understanding of HEV exposure in hemodialysis patients is essential for improving individual patient care and informing broader public health strategies (5).

Among the HEV genotypes that infect humans (1–6), genotypes 1 and 2 are primarily linked to fulminant hepatitis in pregnant women, with maternal mortality rates reaching up to 20% (6, 7). Genotype 3, which is zoonotic, is more frequently observed in industrialized countries and has the potential to cause chronic infections, particularly in immunocompromised individuals. This includes patients with HIV, hematological or oncological malignancies, those receiving immunosuppressive therapy, transplant recipients, and hemodialysis patients (1, 8, 9). Given that genotypes 3 and 4 can be transmitted through blood transfusion and cause outbreaks, screening programs are being developed to enhance protection for high-risk patients (10, 11).

Hemodialysis patients, considered immunosuppressed, are also candidates for kidney transplantation and are at increased risk for bloodstream infections compared to the general population, with risk increasing over time (12). In non-endemic countries where routine HEV screening is not performed, assessing HEV seroprevalence in hemodialysis patients is crucial for mitigating HEV-related complications in this vulnerable group. In line with global variations, HEV seroprevalence studies conducted in Turkey have reported prevalence rates ranging from 3 to 30% across different patient populations and healthy individuals (13, 14).

Few studies have investigated HEV seroprevalence in hemodialysis patients in Turkey. Identifying anti-HEV IgG seropositivity in high-risk groups such as hemodialysis patients can guide targeted screening, improve patient monitoring, and support public health strategies aimed at reducing infection-related morbidity. Therefore, this study aimed to detect anti-HEV IgG antibodies using advanced ELISA assays among asymptomatic hemodialysis patients in Ankara, Turkey. The goal was to obtain reliable epidemiological data and identify potential risk factors associated with HEV transmission.

## Methods

### Study design and patient selection

This study included 160 hemodialysis patients [61 women (38.1%) and 99 men (61.9%)] receiving treatment at three private dialysis centers in Ankara, Turkey, between October and December 2023. Demographic characteristics (age, gender) and potential risk factors for HEV infection—including duration of hemodialysis, comorbidities, residential area (urban or rural), history of blood transfusion, involvement in animal husbandry, drinking water source, and consumption of raw meat—were assessed using survey forms. This was a prospective, open-label study.

### Inclusion and exclusion criteria

The inclusion criteria were:

- Age between 18 and 90 years,
- Currently receiving hemodialysis treatment,
- Providing written informed consent.

The exclusion criteria included:

- Age under 18 or over 90 years,
- Pregnancy.

### Blood sample collection and storage

Blood samples for Anti-HEV-IgG testing were collected during routine dialysis sessions from patients who provided informed consent and met the inclusion criteria. Serum samples were stored at −40°C in a deep freezer until HEV-IgG ELISA testing was performed.

### Anti-HEV-IgG testing procedure

Anti-HEV-IgG testing was conducted using ELISA with an Alisei device (Algen Diagnostik, Italy) and the Dia Pro HEV IgG kit (HEV IgG ELISA, Dia Pro, Italy) according to the manufacturer’s instructions. Serum samples and kit components were prepared at room temperature, and controls and patient samples were processed using specific diluents. Serum samples were obtained by centrifuging whole blood at 3000 rpm for 10 min and were stored at −40°C until analysis. After incubation and washing steps, the enzyme-conjugated antibody and a chromogen/substrate solution were added. The reaction was stopped with sulfuric acid, and absorbance was measured at 450 nm. Results were interpreted semi-quantitatively using S/CO ratios: <0.9 negative, 0.9–1.1 indeterminate, and >1.1 positive. All analyses were performed at the Microbiology Laboratory of Ankara Training and Research Hospital.

### Ethical approval

Ethical approval was granted by the Ethics Committee of Kırıkkale University Faculty of Medicine, and written informed consent was obtained from all patients.

### Statistical analysis

Data analyses were conducted using the Statistical Package for the Social Sciences (SPSS) for Windows, Version 22.0 (SPSS Inc., Chicago, IL, USA). Continuous variables were presented as mean ± standard deviation (SD), median (interquartile range), and minimum–maximum values. Categorical variables were expressed as frequencies and percentages (%). Comparisons of categorical variables were performed using Pearson’s chi-square test or Fisher’s exact test, as appropriate. A *p*-value < 0.05 was considered statistically significant for all analyses.

## Results

The age of the hemodialysis patients ranged from 25 to 74 years, with a mean age of 59.62 ± 10.61 years. Anti-HEV-IgG positivity was detected in 42 patients (26.25%) (Table 1). Of the 42 Anti-HEV-IgG-positive patients, 25 (59.5%) were male, and 17 (40.4%) were female. The mean age of Anti-HEV-IgG-positive patients was 66.19 ± 6.07 years (median: 67.5), which was significantly higher than that of Anti-HEV-IgG-negative patients, who had a mean age of 57.29 ± 10.91 years (median: 60) (*p* < 0.001). Despite this significant age difference, no statistically significant association was found between HEV-IgG positivity and gender (*p* > 0.05) (Table 1).

**Table 1:** Demographical features’ of the participants.

Characteristics	Number of patients (*n*:160)	Anti-HEV-IgG positive patients, *n* (%)	Anti-HEV-IgG negative patients, *n* (%)	*P*-value
Male	99	25 (59.5%)	74 (62.7%)	0.345
Female	61	17 (40.4%)	44 (37.2%)	0.480
Total	160	42 (26.25%)	118 (73.75%)	<0.001
Mean age, years ± SD	59.62 ± 10.61	66.19 ± 6.07	57.29 ± 10.91	<0.001

No statistically significant association was identified between HEV seropositivity and potential risk factors, including comorbidities, residential area (urban or rural), history of blood transfusion, involvement in animal husbandry, source of drinking water, and consumption of raw meat (*p* > 0.05). A detailed comparison of comorbidities and other potential risk factors (residential area, history of blood transfusion, involvement in animal husbandry, source of drinking water, and consumption of raw meat) between Anti-HEV-IgG-positive and -negative patients is provided in Table 2.

**Table 2:** Comparison of comorbidities and risk factors between anti-HEV IgG positive and negative patients.

Comorbid conditions and epidemiological characteristics of the patients		HEV IgG status				*p* value
		Negative		Positive		
		*n*	%	*n*	%	
Comorbidities	Yes	64	54.2%	23	54.8%	0.953
	No	54	45.8%	19	45.2%	
Diabetes mellitus	Yes	43	36.4%	20	47.6%	0.203
	No	75	63.6%	22	52.4%	
Hypertension	Yes	74	62.7%	25	59.5%	0.715
	No	44	37.3%	17	40.5%	
COLD	Yes	3	2.5%	4	9.5%	0.077
	No	115	97.5%	38	90.5%	
Coronary artery disease	Yes	10	8.5%	5	11.9%	0.542
	No	108	91.5%	37	88.1%	
Heart failure	Yes	14	11.9%	3	7.1%	0.563
	No	104	88.1%	39	92.9%	
Residential area	Urban	109	92.4%	38	90.5%	0.745
	Rural	9	7.6%	4	9.5%	
Blood transfusion	Yes	36	30.5%	8	19.0%	0.153
	No	82	69.5%	34	81.0%	
History of animal husbandry	Yes	4	3.4%	2	4.8%	0.653
	No	114	96.6%	40	95.2%	
Source of drinking water	Treated water	24	20.3%	8	19.0%	0.294
	Natural source	36	30.5%	8	19.0%	
	Tap water	58	49.2%	26	61.9%	
Consumption of raw meat	No	118	100.0%	42	100.0%	-

Categorical variables are expressed as frequency (percentage). Chis-quare test or Fisher’s exact test, *p*-value of significance, *p* < 0.05.

The patients were categorized into three age groups: under 55 years, 56–64 years, and over 65 years. The distribution of Anti-HEV-IgG positivity across these age groups is presented in Table 3. A progressive increase in Anti-HEV-IgG seropositivity was observed with advancing age, with a seropositivity rate of 6.7% in patients under 55 years, rising to 47.4% in those over 65 years.

**Table 3:** Anti-HEV-IgG positivity results by age groups in patients.

Comorbid conditions and epidemiological characteristics of the patients		Age groups						*p*
		≤55 year (n:45)		56–64 year (n:58)		≥65 year (n:57)		
		*n*	%	*n*	%	*n*	%	
Anti-HEV IgG results	Negative	42	93.3%	46	79.3%	30	52.6%	<0.001
	Positive	3	6.7%	12	20.7%	27	47.4%	
Gender	Male	27	60.0%	37	63.8%	35	61.4%	0.937

## Discussion

Hepatitis E virus (HEV) poses a significant risk to hemodialysis patients, particularly due to their immunocompromised status. This study investigated HEV-IgG seroprevalence and associated risk factors among hemodialysis patients in Ankara, Turkey. Given Turkey’s strategic location and its variable HEV prevalence, understanding its epidemiology in high-risk populations is critical for designing effective local and regional healthcare responses. Our findings highlight the influence of age, dialysis duration, and environmental factors on HEV transmission. Additionally, comparisons with existing literature reveal regional and international variations, emphasizing the need for further research to refine preventive strategies in this high-risk group.

Globally, approximately 20 million individuals are reported to be infected with HEV annually, with only 3 million cases presenting with significant clinical symptoms. The estimated mortality rate associated with HEV infection ranges between 50,000 and 70,000 deaths per year. Due to the high proportion of asymptomatic infections, accurately determining the true prevalence of HEV remains challenging (15).

Recent HEV-related outbreaks emphasize the importance of understanding real-world epidemiological data, particularly given the risk of viremia progressing to chronic hepatitis and cirrhosis in hemodialysis patients who are candidates for kidney transplantation (16).

Although studies on HEV-IgG seroprevalence in hemodialysis patients in Turkey exist, limited research has focused on the associated risk factors. HEV seroprevalence is known to vary across different regions of Turkey. In a study conducted by Kaleli et al. in Pamukkale, Anti-HEV-IgG positivity was detected in 10 of 94 dialysis patients (10.4%) and in 3 of 32 healthy adults (9.4%) in the control group (17). A meta-analysis by Mızrakçı S. reported regional variations in HEV seroprevalence among hemodialysis patients, with the highest prevalence observed in Central Anatolia (23.4%), followed by Southeastern Anatolia (21.26%), and the lowest in the Aegean region (5.95%). These differences were attributed to socioeconomic factors, hygiene conditions, and dietary habits, particularly the consumption of raw meat products such as raw meatballs (çiğ köfte) (4, 14).

Similar variations in HEV seroprevalence among hemodialysis patients have been reported in other countries. In Bulgaria, Kevorkyan et al. identified an Anti-HEV-IgG positivity rate of 6.2% among 225 hemodialysis patients using the ELISA method (15). A multicenter study conducted in Greece found an average seropositivity rate of 10.4%, with regional variations ranging from 3.8 to 21.7% (13). In Croatia, the seroprevalence among hemodialysis patients was reported at 27.9%, whereas in France and Sweden, the rates were 10.8 and 6%, respectively (16, 18, 19).

In our study, conducted in Ankara, the capital of Turkey and located in Central Anatolia, the HEV-IgG seropositivity rate among hemodialysis patients was 26.25%. This rate is comparable to those reported in Central and Southeastern Anatolia but higher than those observed in the Aegean region and neighboring countries. Logistic regression analysis identified a significant association between hemodialysis duration exceeding five years and an increased risk of HEV infection. Additionally, lower consumption of bottled water was significantly correlated with HEV-IgG seropositivity. These findings underscore the influence of individual patient factors and regional conditions on HEV transmission.

The higher prevalence of HEV in regions where agriculture and animal husbandry are widespread highlights the potential role of zoonotic transmission in HEV epidemiology (20). However, in our study, no statistically significant association was found between the consumption of raw meat, such as raw meatballs (*çiğ köfte*), and Anti-HEV-IgG positivity.

Several studies in Turkey have reported varying HEV seroprevalence rates among hemodialysis patients. In a study by Kılıç et al. (21) conducted in Kayseri, Anti-HEV-IgG positivity was detected in 5.7% of 70 hemodialysis patients and 1.6% of 60 healthy blood donors. Similarly, a study by Uçar et al. in Hatay reported an Anti-HEV-IgG seropositivity rate of 20.6% among 92 hemodialysis patients (22). However, these studies did not identify significant associations between Anti-HEV-IgG positivity and demographic characteristics (age, gender), duration of hemodialysis, history of blood transfusion, or laboratory findings, including serum albumin, platelet count, ALT, and AST levels, or the presence of Anti-HCV and HBsAg positivity.

A study by Sezer et al. reported an Anti-HEV antibody positivity rate of 13.4% in 5 out of 38 hemodialysis patients, with no statistically significant differences observed in HEV seropositivity concerning age, gender, duration of dialysis, history of blood transfusion, volume of blood transfused, ALT levels, or erythropoietin therapy (23). Additionally, a review by Alkan et al. noted that HEV seroprevalence among hemodialysis patients in Turkey was particularly high in the Eastern and Southeastern Anatolia regions, whereas lower prevalence rates were observed in pediatric populations (14).

Our study demonstrated a significant increase in Anti-HEV-IgG positivity with advancing age, with seropositivity rates of 6.7% in patients under 55 years, 20.7% in those aged 56–64 years, and 47.4% in those over 65 years. This age-related increase in seropositivity may reflect cumulative lifetime exposure rather than a current decline in HEV prevalence. It could also be speculated that improvements in sanitation and public health over the past decades have reduced HEV transmission in younger populations, although this trend warrants further investigation (9, 12). Although no statistically significant association was found between HEV seropositivity and gender, the comparable rates across genders within each age group may reflect similar levels of environmental exposure and healthcare-related risk factors, such as dialysis practices, rather than sex-specific susceptibility. Additionally, the elevated HEV seroprevalence among hemodialysis patients may be attributed to their frequent exposure to blood transfusions and the invasive medical procedures inherent in their treatment (4). A meta-analysis by Haffar et al. reported higher HEV-IgG seroprevalence in hemodialysis patients compared to non-dialysis controls (ranging from 0 to 44%), and called for more research on chronic HEV acquisition and its implications—especially in organ transplant candidates who are particularly vulnerable due to immunosuppression (24).

Another study identified advanced age, residence in rural areas compared to urban areas, lower education levels, and the duration of hemodialysis as significant risk factors for HEV seropositivity (25). Consistent with previous findings, our study demonstrated significantly higher Anti-HEV-IgG positivity in older patients. However, no statistically significant associations were found with other risk factors, including blood transfusion, source of drinking water, or consumption of raw meat.

In a study by Mrzljak et al. (16) conducted in Croatia, HEV-IgG seropositivity among hemodialysis patients was higher in those residing in continental regions compared to coastal areas (43.3% vs. 16.8%). While the findings cannot be extrapolated to all of Europe, they underscore potential regional risk differences even within a single country. The higher HEV seroprevalence in Eastern Europe was attributed to the consumption of pork-derived foods. However, the high seroprevalence observed in predominantly Muslim countries such as Turkey (20%), Iran (13.3%), Egypt (22.9%), and Sudan (69%), where pork consumption is religiously prohibited, suggests that alternative transmission routes, including exposure to other animal species such as deer, may play a role (4, 13, 25). In our study, no significant association was found between a history of animal husbandry and HEV seroprevalence. However, given that the study was conducted within a single geographic region, this finding does not provide conclusive evidence against this hypothesis.

The primary risk groups for HEV infection and severe disease include pregnant women, infants, the older adult, immunocompromised individuals, individuals with underlying chronic liver disease, and those in close contact with HEV-infected animals. HEV seroprevalence in Turkey has been reported to range between 3 and 30%, varying according to region and population characteristics (14). Hemodialysis patients represent one of the groups with the highest HEV seroprevalence. In a separate study conducted in Turkey, HEV seroprevalence was reported to be between 13.9 and 20.6% among patients with chronic kidney disease and approximately 35% among agricultural workers (26). This finding suggests that hemodialysis patients face a risk of HEV infection comparable to that of agricultural workers, further reinforcing the role of zoonotic transmission in HEV epidemiology.

A study conducted by Bozdayı et al. in Ankara reported an Anti-HEV antibody positivity rate of 16% among 94 hemodialysis patients, while 44% tested positive for Anti-HCV antibodies (27). Among the Anti-HCV-positive patients, 20% were also positive for Anti-HEV antibodies; however, no significant association was identified between HCV positivity and HEV prevalence. These findings emphasize the importance of identifying risk factors and implementing targeted screening strategies in high-risk populations, such as hemodialysis patients, to enhance the prevention and management of HEV infections.

Although a recombinant capsid-based HEV vaccine has been developed and is available in certain countries, it has not yet been incorporated into routine clinical practice. Prophylactic vaccination may offer protection for naïve travelers and high-risk populations, potentially reducing the burden of HEV infection (1, 3, 28).

### Study limitations

This study has several limitations. First, it was conducted in a single geographic region (Ankara, Turkey), which may limit the generalizability of the findings to other areas with differing socioeconomic and environmental conditions. Second, the cross-sectional study design provides a snapshot of HEV seroprevalence at a single point in time, thereby precluding the establishment of causal relationships between HEV infection and the identified risk factors. Third, although the sample size was adequate for preliminary analyses, future studies with larger, multi-regional cohorts would provide more representative data and improve external validity.

Additionally, risk factor data—including dietary habits, drinking water sources, and involvement in animal husbandry—were self-reported, introducing the possibility of recall bias. Another limitation is the absence of molecular testing, such as HEV RNA detection, which could have provided deeper insights into active infections and chronic cases. Furthermore, the lack of a non-hemodialysis control group limits the ability to draw direct comparisons between hemodialysis patients and the general population. Lastly, the exclusion of specific subgroups, such as pregnant women and individuals under 18 or over 90 years of age, further restricts the generalizability of the findings. Addressing these limitations in future research would enhance the understanding of HEV infection among hemodialysis patients and improve risk assessment and management strategies.

Another limitation is the absence of confirmatory testing such as immunoblot assays to validate ELISA results. Given the potential for false positives with serological assays, especially in immunocompromised populations, the lack of confirmatory testing may have affected the accuracy of seroprevalence estimates (3, 11).

## Conclusion

This study reveals a notable prevalence of Anti-HEV-IgG among hemodialysis patients in Ankara, Turkey, particularly in older adults, highlighting the need for increased attention to this vulnerable group. While most traditional risk factors did not show significant associations with HEV seropositivity, age and extended duration of dialysis emerged as important factors. Given HEV’s ability to cause chronic infections in immunosuppressed individuals, including hemodialysis patients, it is essential to raise awareness among healthcare providers and implement preventive measures such as improved hygiene practices and targeted screenings. Further research involving larger, multi-regional populations is needed to deepen our understanding of HEV’s impact and to guide better prevention and management strategies.

## Data Availability

The raw data supporting the conclusions of this article will be made available by the authors, without undue reservation.
